# A Post-mortem Survey of Bovine Female Reproductive Tracts in the UK

**DOI:** 10.3389/fvets.2019.00451

**Published:** 2019-12-12

**Authors:** Sam Millward, Karin Mueller, Robert Smith, Helen M. Higgins

**Affiliations:** ^1^Oakhill Veterinary Centre, Preston, United Kingdom; ^2^Department of Livestock Health and Welfare, Institute of Veterinary Science, University of Liverpool, Liverpool, United Kingdom

**Keywords:** ovaro-bursal adhesions, infertility, cattle, reproductive tract abnormalities, cystic ovarian disease, anoestrus, post-mortem

## Abstract

The aim of this study was to estimate the prevalence of macroscopic reproductive tract abnormalities in a sample of female cattle in the UK. To our knowledge, this type of post-mortem survey has not been conducted in the UK since the 1970s. Over the last 40 years significant changes have occurred with respect to management and genetics. Moreover, there have been changes in growth rates in beef animals, elevated milk yields and a decline in fertility in dairy cattle. It was hypothesised that differences may exist in the extent and type of lesions occurring compared with previous studies. Between May and July 2017, the reproductive tracts of cattle (*Bos taurus*) were examined post-mortem at an abattoir in the north west of England. All female cattle slaughtered on visit days were eligible. In total 680 tracts were examined, constituting 88% of those eligible. Macroscopic abnormalities were recorded using a standard format and definitions. The majority of cattle were a dairy breed (73%) with Holstein-Friesian accounting for over half of these. Median age at slaughter for dairy breeds was 5.1 years (range 1.7–13.8 years) and 3.9 years (0.92–16.8 years) for beef breeds. A total of 141 out of the 680 reproductive tracts examined exhibited at least one lesion, giving an overall prevalence of abnormalities of 20.7%, with 95% confidence interval (CI) 17.9–23.9%. This is double the last similar UK-based study carried out in the late 1970s. There were 20 different types of abnormality identified, with 207 individual lesions in 141 abnormal tracts. The ovary was the most common anatomical location displaying abnormalities, accounting for 70.2% of all abnormal tracts. Ovaro-bursal adhesions were the most common abnormality found at 5.3% (CI 3.9–7.2%) and half of these were classified as severe. The second most common lesion was follicular cystic ovarian disease at 4.6% (CI 3.2–6.4%), followed by anoestrus at 4.1% (CI 2.9–5.9%). Double the prevalence of macroscopic reproductive tract lesions is a concern. Greater use of post-mortem material for disease surveillance and further studies into risk factors, especially for the most prevalent lesions, is warranted.

## Introduction

Infertility is the most common reason for involuntary culling in UK dairy cattle and is a significant economic drain on the UK cattle industry (1, 2). Infertility and sterility may manifest at a macroscopic level and may be identified on examination of abattoir specimens (3–5). Several abattoir surveys of cattle have taken place around the world previously, with the prevalence of macroscopic reproductive tract abnormalities ranging from 2.8% in Australia (6) to 64.0% in Libya (7), summarised in Mimoune et al. (8).

To the authors' knowledge, however, the last UK-based survey of macroscopic abnormalities of female bovine reproductive tracts was carried out over 40 years ago by Al-Dahash and David (9). Examination of 8,071 tracts revealed an overall abnormality prevalence of 10%, with follicular cystic ovarian disease reported to be the most common finding at 3.8% (9).

In the past four decades since this survey was conducted, genetic improvements in conjunction with the advancement of farm management strategies has significantly enhanced the genotype and phenotypic performance of both dairy and beef breeds of cattle in the UK. In particular, incorporation of the North American Holstein breed into the UK dairy herd in the 1990s has led to a significant increase in milk yields. UK dairy cattle are now producing in the region of double the amount of milk compared to those in the 1970s (10). Furthermore, Wathes et al. (11) have reported that genetic advances in beef animals have resulted in demonstrably higher and more efficient growth rates [summarised in Wathes et al. (11)].

The aim of this study was to start to bridge this knowledge gap by providing an up-to-date estimate of the prevalence of macroscopic reproductive tract abnormalities in a sample of female bovines in the UK. It was hypothesised that a higher prevalence of reproductive tract abnormalities would be found in this study in comparison to the work of Al-Dahash and David (9).

## Materials and Methods

### Study Design

Ethical approval for this cross sectional observational study was granted by the University of Liverpool Veterinary Ethics Committee (Ref. VREC529). Sample size calculations were based on the *a priori* estimate that the proportion (or percentage) of reproductive tracts that were diseased would be between 0.1 and 0.2 (or 10 and 20%). It was desirable to estimate this proportion with a precision of within plus or minus 0.05 (5%), that is 0.1 ± 0.05 (10 ± 5%) or 0.2 ± 0.05 (20 ± 5%). The confidence level for the confidence interval that would be generated by the data was set at 95%. With these values, between 138 and 246 animals were required to be sampled (12).

Post-mortem examination of specimens took place over four non-consecutive days between May and July 2017, at an abattoir in the north west of England, UK. Timing of visits was purely determined by the availability of the first author (Sam Millward) who collected all the data; it was not related to abattoir activities or through-put. All female cattle (*Bos taurus*) slaughtered on the day of the visits were eligible to be enrolled in the study. Cattle of any age, purpose or stage of life cycle could be slaughtered on any day. Reproductive tracts were sampled over the entire duration of the cattle slaughtering shift. Due to the speed of the slaughter line, it was not possible to examine all eligible tracts resulting in some being missed and not examined. This potentially introduced some selection bias, however as there was no reason not to examine any tract except for time constraints, it is the authors' opinion that any systematic bias was minimal.

### Data Collection

A carcass identification number was obtained from the identification tag attached to the carcass, recorded and matched up to the corresponding reproductive tract. Carcass identification tag numbers were cross referenced with abattoir records to obtain the official identity tag number, age, breed, and carcass grade. British Cattle Movement Service (BCMS) data was used to obtain the most recently recorded calving date, where applicable.

Immediately following evisceration, the reproductive tract was separated from the gastrointestinal tract with a knife and removed from the production conveyer belt for detailed examination. All post-mortem examinations of the reproductive tract were carried out by veterinary surgeon Sam Millward.

The external aspects of the vagina, cervix, uterine body, and ovaries were visually examined and palpated and any external abnormalities were recorded. All externally visible ovarian structures were measured using brass vernier calipers to the nearest millimeter. The ovaries were incised longitudinally. The diameter and wall thickness of all corpora lutea and follicles were measured and recorded if ≥25 mm in diameter. The reproductive tract was incised longitudinally from the vagina to the distal tip of each uterine horn for examination of the internal aspect of the reproductive tract and luminal contents.

### Definitions of Macroscopic Abnormalities

Any abnormalities were recorded according to pre-defined definitions, which are described in Table 1 (ovarian abnormalities), Table 2 (uterine abnormalities), and Table 3 (cervical abnormalities). In addition to these, the condition of freemartinism was recorded if the macroscopic appearance of the reproductive tract displayed a combination of the following abnormalities (3, 18): hypoplastic, non-patent vagina; hypoplastic and/or masculinised gonads; hypoplastic or absent Müllerian duct derivatives (vagina, cervix, uterus); visible mesonephric (Wolffian duct) derivatives (vesicular glands/epididymides/ vas deferentia) (22). Oviduct patency was not assessed.

**Table 1:** Definition/description criteria used for abnormalities associated with the ovaries.

**Ovarian abnormality**	**Definition/description criteria**
Anoestrus	Greater than 50 days calved (13) Absence of a corpus luteum (CL), corpus haemorrhagicum, and follicles <25 mm (14) Absence of indicators suggestive of recent oestrus: hyperaemia and oedema of the endometrium; local haemorrhages in the endometrial mucosa; uterine wall pigmentation; a reddish-brown endometrial mucosa; low viscosity mucous; metoestrus bleeding (15)
Follicular cyst	A fluid-filled ovarian structure ≥25 mm in diameter with a wall thickness of <3 mm, in the absence of luteal tissue (3, 16)
Luteal cyst	An ovarian structure ≥25 mm in diameter with a wall thickness ≥3 mm (3, 16). Absence of ovulation papilla (17)
Paraovarian cyst	A fluid-filled structure commonly present in the meso-salpinx; a remnant of the mesonephric duct (3)
Ovaro-bursal adhesions	Adhesions between the ovary and ovarian bursae were classified as: 1. Mild (i.e., fine, fibrous, web-like adhesions) 2. Moderate (i.e., more substantial adhesions with retention of normal anatomical appearance 3. Severe (i.e., extensive adhesions resulting in complete obliteration of normal anatomical appearance)
Hydrosalpinx	Accumulation of fluid adjacent to an occlusion of the oviduct tubal lumen, resulting in distension and thinning of the oviduct wall (3, 18)
Neoplasia	An ovarian structure that appears macroscopically abnormal both externally and internally

**Table 2:** Definition/description criteria used for abnormalities associated with the uterus.

**Uterine abnormality**	**Definition/description criteria**
Pyometra	Distension of the uterine lumen with purulent material in the presence of a CL and a closed cervix (3)
Endometritis	Purulent uterine discharge detected in the vagina ≥21 days after calving. Graded according to the appearance of vaginal mucus in keeping with Sheldon et al. (19) as: Grade 1: mucus containing flecks of white or off-white pus Grade 2: exudate containing <50% white or off-white purulent material Grade 3: exudate containing ≥50% purulent material
Metritis	In a cow with a recently recorded calving date, the presence of fetid brown to red-black uterine luminal contents (17). Uterine mucosa necrotic and haemorrhagic and the wall of the uterus is thickened and oedematous. If severe, fibrin may be present on the serosal surface of the uterus (20).
Segmental aplasia (including unicornis uteri)	Incomplete development of the reproductive tract as a result of defective paramesonephric ducts (Müllerian ducts) (3) Unicornis uteri refers to the presence of only one uterine horn with a normal lumen (3)
Mucometra	Accumulation of clear fluid in the uterine lumen that is not associated with pregnancy (21), with or without the presence of a CL.
Uterine adhesions	Adhesions (or remnants of adhesions) associated with the uterine serosa categorised as: 1. Mild (i.e., fine, fibrous, strand-like adhesions) 2. Moderate (i.e., more substantial fibrous strands, uterine architecture still discernible) 3. Severe (i.e., complete distortion of the normal anatomical appearance of the uterus)
Mummified fetus	Presence of a dead, desiccated fetus in the uterine lumen, surrounded by a viscous chocolate-coloured material in the presence of a CL and a closed cervix (17)
Fetal remnants	The presence of fetal bones within the uterine lumen

**Table 3:** Definition/description criteria used for abnormalities associated with the cervix and vagina.

**Cervix or vagina abnormality**	**Definition/description criteria**
Double cervix	Partial or complete duplication of the cervical canal (18)
Cervicitis	Visual evidence of marked cervical inflammation to include hyperaemia and oedema, with or without a foul odour, lacerations or trauma (17)
Vaginitis	Visual evidence of marked vaginal wall inflammation to include hyperaemia and oedema, in the absence of oestrus, with or without a foul odour, lacerations or trauma (17)
Cervical bands/adhesions	Fibrous adhesions associated with the cervix

### Data Analysis

The BCMS data on breed was used to assign each animal a breed type of either beef or dairy in keeping with Boon (23) (see Table 4). Cross-breeds and pure breeds were classified as a single category. For example, a British Blue cross and a pure British Blue were both classified as a British Blue.

**Table 4:** Allocation of breed type (either dairy or beef) and the number of animals of each breed in the sample population (*n* = 680).

**Dairy**	**Number (%)**	**Beef**	**Number (%)**
Holstein Friesian	358 (52.7)	Limousin	54 (7.9)
Holstein	64 (9.4)	Simmental	26 (3.8)
British Friesian	41 (6.0)	Charolais	17 (2.5)
Brown Swiss	9 (1.3)	British Blue	15 (2.2)
Jersey	9 (1.3)	Hereford	15 (2.2)
Ayrshire	6 (0.9)	Blonde D'Aquitaine	11 (1.6)
Swedish Red	6 (0.9)	Stabiliser	11 (1.6)
Montbeliarde	3 (0.4)	Aberdeen Angus	10 (1.5)
Dairy Shorthorn	2 (0.3)	Shorthorn	9 (1.3)
Danish Red	1 (0.2)	Belgian Blue	5 (0.7)
		Luing	3 (0.4)
		Belted Galloway	1 (0.2)
		Blue Grey	1 (0.2)
		Angler Rotvieh	1 (0.2)
		Red Poll	1 (0.2)
		Salers	1 (0.2)
Total number	499 (73.4)		181 (26.6)

*Percentages are out of 680. The British Friesian is a dual purpose breed which compared to the Holstein dairy breed has a smaller frame size, better fertility and higher body condition score, but lower milk yield. Holstein-Friesian results from cross–breeding Holsteins with Friesians*.

All data was entered into Microsoft® Excel (2016) initially and descriptive statistics and graphs were produced. For the purpose of calculating the overall prevalence of specific abnormalities, an individual reproductive tract that displayed multiple abnormalities of the same type was classified as one abnormality (e.g., a single tract exhibiting three follicular cysts was classified as a single case of follicular cystic ovarian disease).

Minitab® (version 18.1, 2017) was used to perform Pearson's chi-squared tests between the categorical variables breed type (dairy/beef), abnormal reproductive tract (yes/no: both overall, and for specific conditions) and age category (≤30 or >30 months). A Mann-Whitney test was also used to determine whether the median age of animals with and without reproductive tract abnormalities differed. Statistical significance was defined as *p* < 0.05. Wilson score interval (24) was used to calculate 95% confidence intervals for prevalence which are reported in brackets following point estimates.

## Results

### Demographics

Post-mortem examination was carried out on 680 of a possible 775 eligible female reproductive tracts from cattle slaughtered over the duration of time spent in the abattoir (88% of all possible eligible specimens examined). Almost three-quarters of cattle were of a dairy breed (73%) with Holstein-Friesian cattle accounting for over half of all cattle (53%). In total, 26 different breeds were examined (see Table 4).

**Figure 1:**
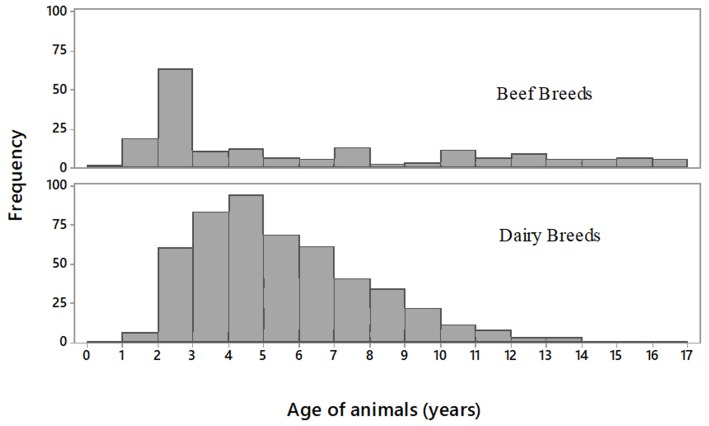
Histograms showing the age distribution of the sample population (*n* = 680 in total) grouped by beef breeds (*n* = 181) and dairy breeds (*n* = 499).

Figure 1 shows the age distribution of animals, grouped by breed type. The median, mean and standard deviation of age at slaughter (in years) for beef breeds was 3.9, 6.0, and 4.7. For dairy breeds these figures were 5.1, 5.5, and 2.4.

### Estimated Prevalence of Female Macroscopic Reproductive Tract Abnormalities

Of the 680 reproductive tracts examined, 141 tracts presented with at least one macroscopic abnormality, i.e., the overall prevalence of abnormalities was estimated to be 20.7% (17.9–23.9%). A total of 207 individual lesions were detected in these 141 abnormal tracts.

**Figure 2:**
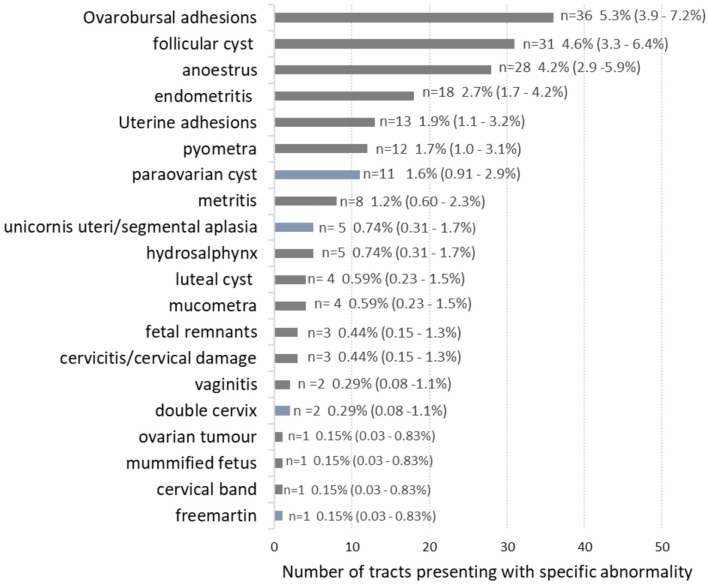
The number of female bovine reproductive tracts exhibiting specific abnormalities. Blue bars highlight congenital lesions, grey bars are acquired lesions. In total, there were 141 individual tracts with abnormalities, but because some tracts had more than one type of abnormality the numbers in the figure tally to 189. Prevalence as a percentage of all tracts examined (*n* = 680) with 95% confidence interval in brackets.

Of all tracts examined, ovaro-bursal adhesions were the most common abnormality found (36/680) giving an estimated prevalence of 5.3% (3.9–7.2%), followed by follicular cysts at 4.6% (3.2–6.4%) and anoestrus at 4.1% (2.9–5.9%). See Figure 2 for details of other abnormalities. Of the 141 abnormal tracts, 100 tracts had a single lesion, 25 tracts had 2 lesions, 9 tracts had 3 lesions, 6 tracts had 4 lesions, and 1 tract had 6 lesions.

Out of the abnormal tracts, acquired abnormalities were much more common than congenital abnormalities. Thus, 122 out of 141 abnormal tracts had only acquired lesions (86.5%), 10 tracts had only congenital lesions (7.1%) and 9 tracts had both congenital and acquired lesions (6.4%).

### Anatomical Location and Severity of Abnormalities

The most common anatomical location to present with abnormalities was the ovary, such that 77 out of the 141 abnormal tracts (54.6%) only had a lesion associated with the ovary/ovaries. This was followed by the uterus, with 36/141 (25.5%) abnormal tracts only having a uterine issue. There were 21/141 abnormal tracts (14.9%) with lesions located in both the ovary and uterus, 5/141 abnormal tracts with cervix or vagina lesions, 1 abnormal tract with uterus, cervix and vagina lesions, and 1 abnormal tract with ovary, uterus and cervix/vagina lesions.

Of all abnormal tracts with ovarian abnormalities, including anoestrus, the most common presentation was bilateral (42/99; 42.4%), followed by right unilateral (35/99; 35.4%) and left unilateral (22/99; 22.2%). A total of 41 individual ovaro-bursal adhesions were found in 36 tracts. These were categorised as mild (*n* = 22), moderate (*n* = 1), and severe (*n* = 18). A total of 44 individual follicular cysts were identified in 31 tracts; 19 of these tracts had a single cyst, 11 tracts had 2 cysts and one tract had 3 cysts. The 4 single luteal cysts identified where in 4 separate tracts. The 18 cases of endometritis were categorised as grade 1 (*n* = 7), grade 2 (*n* = 8), and grade 3 (*n* = 3).

### Prevalence of Reproductive Tract Abnormalities by Age and Breed Type

The overall odds of a dairy animal presenting with an abnormal tract was 1.6 times the odds of a beef animal presenting with an abnormal tract: 22.6% (113/499) for dairy breeds vs. 15.5% (28/181) for beef breeds (*p* < 0.05). Breaking this down by age category revealed an even higher odds (3.9 times the odds) of a young dairy animal (i.e., ≤30 months old) having an abnormal tract compared to a young beef animal: 33.3 vs. 11.3% (*p* < 0.05). As per Figure 1, a far higher proportion of beef animals were seen in the young age category of 0–30 months: 34.2% of all beef animals (62/181) compared to 4.8% (24/499) of all dairy animals. None of the beef animals ≤30 months old had a recorded calving date, compared with 5 (of the 24) dairy animals ≤30 months of age.

A higher prevalence of abnormalities in older dairy animals (>30 months of age) was noted compared with older beef animals, although this was not statistically significant (22.1 vs. 17.6% (*p* = 0.29). No statistically significant associations in the prevalence of *individual* abnormalities were seen between dairy and beef breeds. There was also no statistically significant difference between the median age of animals with abnormal reproductive tracts (4.75 years) compared to those with normal tracts (4.83 years) *p* = 0.84.

## Discussion

### Overall Prevalence of Female Bovine Reproductive Macroscopic Abnormalities

We found approximately double the prevalence of abnormalities (20.7%; CI 17.9–23.9%) since the last UK abattoir survey over 40 years ago (9), providing some support for the hypothesis that the prevalence has increased over time. The study by Al-Dahash and David (9) did not include confidence intervals for their point estimate of 9.96%. However, retrospective analysis of their raw data (804/8071 abnormal tracts) using a Wilson score interval results in a 95% confidence interval of 9.3–10.6%.

In agreement with Noakes et al. (3), acquired abnormalities were found to be more common than congenital abnormalities in this study. Part of the explanation for this could be related to immune dysfunction and physiological disturbance attributable primarily to increased metabolic stress in dairy cattle. Moreover, congenital genetic abnormalities that reduce functionality do not get passed on and increase in the population over time.

Compared with the findings of Al-Dahash and David (9), a higher prevalence of both congenital and acquired abnormalities were recorded. If this is a true reflection, speculation also arises as to whether genetics or other specific environmental exposures may be contributing to the higher prevalence of congenital abnormalities observed today. However, comparisons are limited as the authors did not provide details of the age distribution or breed type of the animals examined. It is worth noting that overall, a less extensive list of different types of abnormalities was described by Al-Dahash and David (9) compared with our findings. This may reflect a genuine difference in the sample populations, or possibly missed observations.

### Ovaro-Bursal Adhesions

The most prevalent abnormality found in this study was ovaro-bursal adhesions (5.3%). This is comparable to the findings of a UK study of uterine tube abnormalities by Kessy and Noakes (25) who found a prevalence of 6.9%. A total of 18 tracts exhibited severe ovaro-bursal adhesions in the present study, accounting for 2.6% (13/680) of the total number of tracts examined. This is higher when compared to Kessy and Noakes (25) in which 1.8% (36/2000) of specimens presented with “extensive ovaro-bursal adhesions”. Unfortunately oviduct patency was not established in the current study due to time constraints.

Consistent with the findings of Al-Dahash and David (9), we found evidence of ovarian cyclicity despite the presence of severe ovaro-bursal adhesions. It is thought that severe ovaro-bursal adhesions impair fertility through physical obstruction of the infundibulum leading to compromised oocyte transport or physical interference with ovulation (25). It is likely, therefore, that the four cows that presented with bilateral severe adhesions in this survey were sterile. It is assumed that severe ovaro-bursal adhesions are permanent owing to the generation of significant quantities of fibrotic tissue.

Many mild “ovulation tag” adhesions are thought to resolve spontaneously, as they appear to be more common in cyclical heifers than in mature cows (18). Mild lesions are thought to originate from the blood clots and follicular fluid released at ovulation, as supported by their absence in prepubertal heifers (18). Noakes et al. (3) refer to these lesions as “physiological hazards”. These minor lesions are thought to bear little significance with respect to fertility and cases have been found in association with pregnancy (25, 26).

Some suggested aetiologies of moderate to severe ovaro-bursal adhesions include excessive haemorrhage following ovulation, trauma on rectal palpation/parturition, generalised peritonitis, manual rupture of follicular cysts, uterine irrigation with irritant substances and ascending uterine infections (3, 18, 25).

#### Cystic Ovarian Disease (COD)

As in this study, Al-Dahash and David (9) used a diameter threshold of ≥25 mm to define a cystic structure. However, Al-Dahash and David (9) did not quantify cyst wall thickness; merely classifying them as either “thick” or “thin” walled cysts. Therefore, the prevalence of the different types of ovarian cysts recorded in 1977 cannot be reliably compared to the findings of this study. A higher overall combined prevalence of follicular and luteal COD was found in this study compared with the combined prevalence of “thick” and “thin” walled cysts in 1977. A higher prevalence of follicular COD was noted in dairy animals compared with beef animals in the present study, although this was not statistically significant.

Milk yields have doubled since the 1970's (10). Increased milk yields are thought to predispose the development of COD due to the negative energy balance-mediated disturbance of both follicular growth and hypothalamic-pituitary-gonadal axis function (16). A genetic correlation is also known to exist between COD and milk yield (16). Over the years, intense genetic selection for increased milk yields provides a possible explanation behind the increased prevalence of COD seen compared to the relatively low production animals in the 1970s. There is also the possibility that for some luteal cysts they are normal corpus luteum with a lacuna.

#### Anoestrus

Diagnosis of anoestrus at post-mortem poses a challenge as ovarian cyclicity is a dynamic process. Animals may be misdiagnosed as anoestrus post-ovulation prior to the formation of a CL (14). In live animals, the use of a vaginal speculum, transrectal ultrasound or re-examination after 7–10 days has been recommended in cases of an uncertain diagnosis (27, 28). Detailed examination of the uterus and vagina for macroscopic changes suggestive of recent oestrus were used in this study to try to avoid the misclassification of uteri examined in the immediate post-ovulation stage and to aid confirmation of anoestrus (15).

We identified a total of 4.1% of tracts (28/680) with anoestrus. No meaningful comparisons could be made to Al-Dahash and David (9), as they did not report any cases of anoestrus. Globally, 1.95–22% of post-mortem specimens have been classified as anoestrus (8, 29, 30). In contrast to this study, some of these previous studies fail to take into consideration all potential macroscopic indicators of ovarian cyclicity, therefore the prevalence of anoestrus may have been overestimated.

Physiological disturbance of the hypothalamic-pituitary-gonadal axis may manifest as anoestrus (14). Stressors, such as negative energy balance, malnutrition, and lameness are known to have adverse effects on GnRH/LH pulse frequency and the subsequent capacity to stimulate an LH surge and ovulation (31, 32). This raises the question as to what specific stressors are causing anoestrus in the specimens seen in this study. High ambient temperatures at the time the study was carried out and/or transport and mixing of animals on collection farms and markets in the days leading up to slaughter may have contributed to stress-induced hypothalamic-pituitary-gonadal axis dysfunction, manifesting macroscopically as anoestrus or COD (33). However, we do not have any data concerning how long animals in our study had been away from their home farm before slaughter. Extending post-mortem evaluation beyond the reproductive tract may have provided some evidence for potential stressors that may have contributed to reproductive dysfunction.

### Differences Between Dairy and Beef Breeds

The higher overall prevalence of reproductive tract abnormalities in dairy breeds compared to beef breeds appeared to have been influenced by the population age demographics, with young (≤30 month old) dairy animals far more likely to have abnormalities, compared to young beef animals. It seems likely that this difference reflects production purpose: beef heifers are slaughtered at a young age for beef production or, if retained for breeding, may have a later target for age at first calving, while dairy heifers generally are retained for breeding and milk production, only being culled if they do not conceive. Thus, the dairy heifer population may be selected for low fertility and one of the possible causes is reproductive tract abnormalities. Therefore, it is likely that a higher proportion of the young beef animals in our sample would not have been bred, nor experienced parturition or lactation, compared to the dairy heifers. Parturition is associated with a risk of physical trauma to the tissues of the reproductive tract. In addition to compromised integrity of anatomical reproductive tract barriers (vulva, vagina, cervix and endometrium), periparturient metabolic stress and immunosuppression increase the risk of acquisition of macroscopic reproductive tract abnormalities (34, 35). It is well documented that negative energy balance and uterine infection are deleterious to the physiological re-establishment and function of the hypothalamic-pituitary-gonadal axis (31, 36). This line of reasoning is supported by the fact that none of the 62 beef animals ≤30 months of age in our study had a recorded calving date or were diagnosed with a case of uterine infection, whilst 5 of the 24 dairy animals ≤30 months of age had a recorded calving date and 3 were diagnosed with uterine infection.

Similar conclusions have been drawn by Mylrea (26), who found a higher incidence of genital tract abnormalities in dairy cattle (23.7%) compared with beef cattle (11.5%). Mylrea (26) attributed the difference in the prevalence of abnormalities to the fact that within the sample population, the beef animals were much younger (many maiden heifers) and the dairy animals were older, mature cows.

### Interpretation of Abnormalities and Limitations

Macroscopic abnormalities of the reproductive tract may be permanent or transient, depending upon the nature of the abnormality, the efficacy of any therapeutic interventions and the time period elapsed between development of the abnormality and slaughter. The significance of individual abnormalities with respect to fertility varies from being inconsequential to resulting in permanent sterility (3). Not all macroscopic abnormalities can be assumed to have an adverse effect on fertility, particularly if the breeding history is unavailable, as was the case in this study. It is also possible that some abnormalities found at slaughter may have arisen following cessation of breeding attempts and were not directly involved in reproductive failure. Furthermore, some causes of infertility do not present on a macroscopic level, for instance subclinical endometritis (34, 37). Ansari-lari et al. (38) found that farmers tended to keep infertile cows for longer periods from calving to culling, compared with cattle culled for other reasons. Based on this, it is also possible that some reversible acquired macroscopic abnormalities that may have contributed to reproductive failure, could have resolved at a macroscopic level by the time of culling. These factors should be kept in mind when considering our findings.

In addition, as noted above, differences in study design, availability of relevant information required to interpret observations (e.g., calving dates), and in some cases lack of precise information regarding definitions of lesions, make it more challenging to make direct comparisons between our findings and previous work. Furthermore, it should be kept in mind that this survey took part at a single abattoir in the north-west of the UK. While this is a relatively cattle-dense area, nevertheless due caution should be taken when attempting to generalise these findings to any wider populations. According to UK slaughter statistics (39), 56,972 heifers and 47,335 cows were slaughtered during the month of June 2017, and these figures appear typical for the UK, although breed information is not available. Comparing this to the age distribution of animals in our study, it seems likely that we had a higher proportion of cull cows, compared to all UK slaughtering (only 13% of animals in our sample were ≤30 months old).

### Considerations for the Future

In the authors' opinion, abattoir and knacker-yard post-mortem material is generally an under-utilised resource for disease surveillance. In the face of diminishing fertility, we advocate that similar surveillance surveys should be carried out more frequently in the future. Condemnation of animals with fertility-compromising macroscopic reproductive tract abnormalities may be disregarded on an individual animal level or even remain undiagnosed. Feedback of information to veterinary surgeons and breeding companies, at both a herd and a national level, could provide important and relevant information. If further risk factors could be identified and linked to the development of specific fertility-compromising acquired or congenital macroscopic abnormalities, it may be possible to implement changes to minimise these risk factors, through targeted genetic selection or alternative herd management.

Future studies could include post-mortem findings of the whole carcass. This may help to establish if any associations exist between specific macroscopic reproductive tract abnormalities and other systemic/localised macroscopic abnormalities. It is important that future studies can be more reliably cross-compared. This requires sample population demographics to be reported and abnormalities to be very carefully defined and definitions used reported. In view of improving reproductive performance in UK cattle, it would be of particular interest to assess infertility culls in comparison to cattle culled for reasons other than infertility.

## Conclusion

A total of 141 out of the 680 female bovine reproductive tracts examined exhibited at least one macroscopic lesion, giving an overall prevalence of abnormalities of 20.7%. This is approximately double the last similar UK-based abattoir survey carried out by Al-Dahash and David (9). There were 20 different types of abnormality identified, with 207 individual lesions in the 141 abnormal tracts. The ovary was the most common anatomical location displaying abnormalities, accounting for 70.2% of all abnormal tracts. Ovaro-bursal adhesions were the most common abnormality found at 5.3%. This was closely followed by follicular cystic ovarian disease at 4.6% and anoestrus at 4.1%. Double the prevalence of macroscopic reproductive tract lesions is a concern. Greater use of post-mortem material for disease surveillance and further studies into risk factors, especially for the most prevalent lesions, is warranted.

## Data Availability Statement

The datasets generated for this study are available on request to the corresponding author.

## Ethics Statement

The animal study was reviewed and approved by University of Liverpool Veterinary Ethics committee (Reference number VREC529), Institute of Veterinary Science, Leahurst Campus, University of Liverpool, Neston, Cheshire, United Kingdom.

## Author Contributions

SM conceived the original idea for the study and collected the data. SM and HH analysed the data. SM wrote the first draft of the manuscript, which HH edited. All authors contributed to study design, manuscript revision, read, and approved the submitted version.

### Conflict of Interest

The authors declare that the research was conducted in the absence of any commercial or financial relationships that could be construed as a potential conflict of interest.
